# Editorial: Targeted immunological therapies in dermatology

**DOI:** 10.3389/fmed.2024.1428277

**Published:** 2024-05-15

**Authors:** Yingrou Tan, Nakamizo Satoshi, Hong Liang Tey

**Affiliations:** ^1^National Skin Centre, Singapore, Singapore; ^2^Skin Research Institute of Singapore (SRIS), Agency for Science, Technology and Research (A^*^STAR), Singapore, Singapore; ^3^Graduate School of Medicine, Kyoto University, Kyoto, Japan; ^4^Lee Kong Chian School of Medicine, Nanyang Technological University, Singapore, Singapore

**Keywords:** targeted, immunotherapy, dermatology, pathogenesis, efficacy, safety, toxicity

Cutaneous inflammation, particularly for chronic inflammatory diseases such as eczema and psoriasis are traditionally treated with corticosteroids to control ongoing inflammation. However, prolonged usage of steroids can lead to various side-effects such as thinning of skin, stretch marks or easy bruising. Prolonged suppression of the immune system may also result in secondary infections, driving more inflammation. Next-generation targeted immunological therapies can thus provide an alternative way for us to treat cutaneous inflammation in a more precise manner; these therapies are often wide-ranging in nature, encompassing biologics, small-molecule inhibitors, or RNA-based therapeutics.

Our Research Topic has two key focuses: firstly, we explore how the immune environment and response could contribute to disease pathogenesis; secondly, we dive into the different immunotherapies available for cutaneous diseases, and their associated efficacies, toxicities, and safety profiles.

In the first half of the topic, Chen et al. review how interplay between keratinocytes, the epithelial immune microenvironment and nerves may contribute to psoriasis pathogenesis. Separately, Yamamura et al. also undertake a comprehensive review of cytokines involved in pathogenesis of atopic dermatitis in both mice and humans, simultaneously taking into account publicly available negative clinical trial data to evaluate the importance of certain cytokines clinically.

Wang et al. further explore how actinic keratosis can be caused by factors such as chronic inflammation, oxidative stress, immunosuppression and human papillomavirus infection in addition to mutagenesis and prolonged ultraviolet radiation exposure. At the same time, Lim et al. use *in vivo* optical coherence tomography imaging of patients suffering from idiopathic itch to identify the cause as partial sweat duct obstruction, which resolves partially with retinoid treatment.

In the second half, the articles delve into the plethora of immunotherapies available for hair loss for alopecia areata and androgenetic alopecia; psoriasis and melanoma, discussing the associated efficacies, toxicities, and safety profiles. Hair loss is associated with collapse of immune privilege of the hair follicle, and Toh and Wang discuss how immunosuppression using Janus kinase (JAK) inhibitors, statins and a low dose of interleukin-2 to expand T regulatory populations can be used to restore immune privilege in the hair follicle. Identifying latent infections such as tuberculosis, hepatitis or human immunodeficiency in patients suffering from alopecia areata is also crucial prior to initiating JAK inhibitor treatment, and Huang et al. conduct a retrospective screening study in Changsha, China to identify understand the frequency of such infections in the patient population.

To evaluate the efficacy of different immunotherapies for genital warts, condyloma acuminatum, which arises from human papillomavirus infection, Liu and Qi carry out a network meta-analysis comparing 8 different randomized controlled trials, subsequently concluding that treatment with the Bacillus Calmette-Guerin (BCG) vaccine is most efficacious, which could be due to the induction of the T helper 1 response to BCG which can aid in eliminating viral infection. Simultaneously, Ito et al. carry out a single-center retrospective study in Japan to understand how safe extended interval dosing of anti-programmed death-1 (PD-1) therapy is in Asian patients with melanoma by analyzing the immune-related adverse events. The long-term efficacy and safety of anti-interleukin-23 monoclonal antibody, guselkumab, for plaque psoriasis treatment was also assessed in a single-center retrospective trial in Chinese patients by Zheng et al.. Lastly, EGFRI are likely to induce skin irritation in the form of papulopustular rash through increased cytokine secretion to induce an inflammatory infiltrate. Hence, to obtain a better understanding of cutaneous toxicity associated with epidermal growth factor receptor inhibitors (EGFRI), Dan et al. carried out a disproportionality analysis on data from the FDA adverse event reporting system database (FAERS) for different types of EGFRIs and found that most adverse events occur within the first few days to 2 months.

This Research Topic highlights the transformative potential of targeted immunological therapies in treating cutaneous diseases. By delving into the mechanisms of disease pathogenesis and exploring innovative treatments like JAK inhibitors and monoclonal antibodies, the featured research underscores a shift toward more precise and effective interventions. These advancements promise not only reduced side effects but also enhanced efficacy in certain conditions, heralding a new era in dermatological care.

## Author contributions

YT: Writing—review & editing, Writing—original draft. NS: Writing—review & editing. HLT: Writing—review & editing, Supervision, Resources, Funding acquisition.

